# Targeting phosphoglycerate dehydrogenase in multiple myeloma

**DOI:** 10.1186/s40164-020-00196-w

**Published:** 2021-01-04

**Authors:** Samah Elsaadi, Ida Steiro, Pegah Abdollahi, Esten N. Vandsemb, Rui Yang, Tobias S. Slørdahl, Torstein Baade Rø, Eline Menu, Anne-Marit Sponaas, Magne Børset

**Affiliations:** 1 Center for Myeloma Research, Department of Clinical and Molecular Medicine, Faculty of Medicine and Health Sciences, Norwegian University of Science and Technology (NTNU), Prinsesse Kristinas gate 1, 7030 Trondheim, Norway; 2 Laboratory Clinic, St. Olavs University Hospital, Trondheim, Norway; 3 Clinic of Medicine, St. Olavs Hospital, Trondheim University Hospital, Trondheim, Norway; 4 Children’s Clinic, St. Olavs Hospital, Trondheim University Hospital, Trondheim, Norway; 5 Department of Hematology and Immunology, Myeloma Center Brussels, Vrije Universiteit Brussel (VUB), 1090 Brussels, Belgium; 6 Department of Immunology and Transfusion Medicine, St. Olavs Hospital, Trondheim University Hospital, Trondheim, Norway

**Keywords:** Myeloma, PHGDH, Serine, NCT-503, Bortezomib

## Abstract

**Background:**

Multiple myeloma (MM) is a hematological malignancy characterized by the clonal expansion of plasma cells in the bone marrow. To date, this disease is still incurable and novel therapeutic approaches are required. Phosphoglycerate dehydrogenase (PHGDH) is the first and rate-limiting enzyme in the de novo serine synthesis pathway, and it has been attributed to bortezomib-resistance in MM.

**Methods:**

Two different PHGDH inhibitors, CBR5884 and NCT-503, were tested against human myeloma cell lines, primary MM cells from patients, and peripheral blood mononuclear cells isolated from healthy donors. The PHGDH inhibitors were then tested in combination with proteasome inhibitors in different MM cell lines, including proteasome-resistant cell lines. Furthermore, we confirmed the effects of PHGDH inhibition through knocking down PHGDH and the effect of NCT-503 in vivo in the 5T33MM mouse model.

**Results:**

All the tested myeloma cell lines expressed PHGDH and were sensitive to doses of NCT-503 that were tolerated by peripheral blood mononuclear cells isolated from healthy donors. Upon testing bortezomib in combination with NCT-503, we noticed a clear synergy in several HMCLs. The sensitivity to bortezomib also increased after PHGDH knockdown, mimicking the effect of NCT-503 treatment. Interestingly, targeting PHGDH reduced the intracellular redox capacity of the cells. Furthermore, combination treatment with NCT-503 and bortezomib exhibited a therapeutic advantage in vivo*.*

**Conclusions:**

Our study shows the therapeutic potential of targeting PHGDH in MM, and suggest it as a way to overcome the resistance to proteasome inhibitors.

## Background

Multiple myeloma (MM) is a hematological malignancy with clonal expansion of plasma cells infiltrating the bone marrow (BM), and it accounts for approximately 10% of all hematological malignancies [1]. MM is characterized by the secretion of monoclonal immunoglobulin, known as M-protein. The clinical features associated with MM include osteolytic bone disease, hypercalcemia, renal dysfunction, and anemia. Average life expectancy of MM patients after diagnosis has nearly doubled in the last two decades due to novel drugs, namely immune modulators (IMIDs), proteasome inhibitors (PIs), and monoclonal antibodies, and has reached 5 to 7 years. However, MM is still incurable with high relapse frequency, mainly due to the development of resistance towards available treatments, such as the PIs [2, 3].

Proteasomes are protein complexes that form a ubiquitin-dependent protein degradation system [4]. Since most myeloma cells produce large quantities of immunoglobulins, it is believed that they produce many misfolded proteins [5]. Therefore, an efficient protein degradation system is necessary to maintain cell homeostasis. This makes MM cells especially vulnerable to the use of PIs, such as carfilzomib and bortezomib.

Metabolic reprogramming is a hallmark of cancer, and metabolic alterations have a vital role in cell survival and development of drug resistance also in multiple myeloma [6, 7]. Interestingly, in a study comparing the gene expression in cancer cells from MM patients with cancer cells from two related B cell cancers, chronic lymphocytic leukemia and Waldenström’s macroglobulinemia, phosphoglycerate dehydrogenase (PHGDH) was ranked number 15 on the list of upregulated genes in MM, indicating that this enzyme is of particular significance in MM [8].

PHGDH is the first and rate-limiting enzyme in the de novo serine synthesis pathway (SSP). Two PHGDH subunits oligomerize to convert 3-phosphoglycerate to phosphohydroxypyruvate in an NAD^+^-dependent reaction. This pathway is important for the production of serine and glycine, the latter being an important source of one-carbon units for purines, thymidine, and glutathione (GSH) synthesis [9, 10]. Serine is a conditionally essential amino acid that can be synthesized from glucose or imported from the surroundings, depending on the cellular demand. However, some cancerous cells require an active SSP even when extracellular serine is abundant [11, 12]. Recently, the SSP has been highlighted as a contributor to bortezomib resistance in MM [13]. Therefore, we decided to test the therapeutic potential of targeting PHGDH in MM.

## Methods

### Cell culture

The human myeloma cell lines (HMCLs) used were cultured in a humid atmosphere consisting of 5% CO_2_ at 37 °C with different media depending on the cell line and the use of the cells (Table 1). New cells were thawed at least every 4 months from vials aliquoted with cells propagated shortly after receiving the cells from their described original source, and they were regularly tested to ensure the absence of *mycoplasma*. The HMCLs are from different sources:

- IH1, OH2 and KJON1: in-house cell lines established at St. Olavs University Hospital, Trondheim, Norway [14–16].
- INA6: kind gift from Dr. M Gramatzki, University of Erlangen-Nurnburg, Erlangen, Germany [17].
- ANBL6: kind gift from Dr. D Jelinek, Mayo Clinic, Rochester, MN, USA [18].
- RPMI8226 and U266: from America Type Culture Collection (ATCC), Rockville, MD, USA [19, 20].
- JJN3: Kind gift from Dr. I.M. Franklin, University of Birmingham, Birmingham, UK [21].
- AMO1: Kind gift from the lab of Dr. Christoph Driessen at St. Gallen, Switzerland [22].

**Table 1:** The growth and experiment media for HMCLs

HMCLs	Growth medium	Experiment medium
IH1 and OH2	10% HS in RPMI + IL-6 (sup.)	2% HS in RPMI + IL-6 (1 ng/ml)
INA6 and ANBL6	10% FCS in RPMI + IL-6 (1 ng/mL)	2% HS in RPMI + IL-6 (1 ng/ml)
KJON1	5% HS in RPMI + IL-6 (sup.)	2% HS in RPMI + IL-6 (1 ng/ml)
JJN3 and AMO1	10% FCS in RPMI	2% HS in RPMI
RPMI8226	20% FCS in RPMI	2% HS in RPMI
U266	15% FCS in RPMI	2% HS in RPMI

IL-6 (Gibco^®^ by life technologies™)

IL-6 sup: Cytokine mixture that includes IL-6, produced by monocytes upon activation with lipopolysaccharide

*HS* heat-inactivated human serum (Blood Bank, St. Olav's Hospital), *FCS* Fetal calf serum (Gibco® by life technologies™), *RPMI* RPMI 1640 supplemented with 0.68 mM of L-glutamine (both from Sigma-Aldrich, St. Louis, MO, USA)

### Primary cells

To obtain primary myeloma cells, CD138+ cells were isolated from BM specimens obtained through the Norwegian Myeloma Biobank using RoboSep automated cell separator and Human CD138 Positive Selection Kit (StemCell Technologies, Grenoble, France). Informed consent was obtained from participating patients, and the regional ethics committee approved the study (REK – nr: 2011/2029). These cells were kept in 2% HS in RPMI + IL-6 (sup.). Peripheral blood mononuclear cells (PBMCs) were obtained from EDTA‐blood from healthy controls by density gradient centrifugation using Lymphoprep (Axis‐Shield, Oslo, Norway) and kept in 2% HS in RPMI.

### Transduction

Following the manufacturer’s protocol, INA6 knockdown cells (INA6-KD) were transduced with lentiviral particles containing either non-target control shRNA (shCTR) or shRNA targeting PHGDH (shPHGDH) (Santa Cruz Biotechnology, Dallas, TX, USA; sc-108080 and sc-105011-V). Puromycin (0.25 μg/ml) (Millipore, Burlington, MA, USA) was added to the growth medium as a selection agent.

### Proteasome inhibitor-resistant cells

PI-resistant (carfilzomib-resistant [CR] and bortezomib-resistant [BR]) and control (CTR) clones of two MM cell lines, INA6 and AMO1, were used. For each of the cell lines the three clones combined will further be referred to as INA6-res and AMO1-res. The PI-resistant clones of INA6 and AMO1 were adapted to tolerate higher doses of PIs through gradually increasing the PI concentrations in their growth media. The CTR clones had been cultivated alongside the PI-resistant clones without any drugs [22–24].

### Cell viability assay

Annexin-V-fluorescein isothiocyanate (FITC) kit (Tau Technologies, Albuquerque, NM, USA) was used to determine cell viability. 50 000 cells were seeded in 96-well plates, treated as indicated overnight, before incubating them with annexin V FITC (0.2 μg/ml in 1× annexin binding buffer) for 1 h on ice. Propidium iodide (1.4 μg/ml) was added 5 min prior to data acquisition using an LSRII flow cytometer (BD Biosciences, San Jose, CA, USA). FlowJo Software v10.1 was used to perform the data analysis. Cells negative for both annexin V and propidium iodide staining were considered viable.

### Cell proliferation assay

CellTiter-Glo (CTG) (Promega, Madison, WI, USA) was used to determine cell proliferation. 10,000 cells were seeded in 96-well plates and treated as indicated, before measurement of cell proliferation according to the manufacturer’s protocol. The readout is Relative Luminesence Unit (RLU) and was determined with a Victor 1420 multilabel counter (Perkin Elmer Inc., Waltham, MA, USA). All conditions were done in at least duplicates, and all experiments were performed at least three times.

### Glutathione and NADPH/NADP^+^ assay

GSH-Glo Glutathione Assay and NADP/NADPH-Glo Assay (both from Promega) were used to measure the levels of GSH and NADPH/NADP^+^ (total and ratio), respectively, following the manufacturer’s instructions.

### Immunoblotting

Cells were washed with ice-cold phosphate-buffered saline (PBS) and lysed in lysis buffer: 1% of IGEPAL^®^ CA-630 (Sigma-Aldrich), 150 mM NaCl, 50 mM Tris–HCl pH 7.5, 10% glycerol, 50 mM NaF, 1 mM Na_3_VO_4,_ and a protease‐phosphatase inhibitor mixture (Complete mini tablets; Roche, Basel, Switzerland). After 30 min on ice, the cell debris was pelleted by centrifugation at 12,000*g*, 4 °C for 10 min. Protein concentrations were measured using Quick Start™ Bradford 1× Dye Reagent (Bio-Rad, CA, USA) and iMark™ Microplate Reader (Bio-Rad). Afterward, the samples were diluted to similar concentration and mixed with lithium dodecyl sulfate sample buffer (Invitrogen, CA, USA) with 10 mM dithiothreitol, heated for 10 min at 70 °C and separated on 4–12% Bis‐Tris gels with MOPS running buffer (Invitrogen). Proteins were then transferred to a nitrocellulose membrane using the iBlot Dry Blotting System (Invitrogen). Membranes were blocked with 5% BSA in Tris‐buffered saline with 0.05% Tween20 and incubated with antibodies against indicated proteins overnight at 4 °C. Detection was performed with horseradish peroxidase‐conjugated antibodies (DakoCytomation, Copenhagen, Denmark) and Supersignal West Femto Maximum Sensitivity Substrate (Thermo Fisher Scientific, Rockford, IL, USA). Images were acquired with Odyssey Fc imager (LI-COR Biosciences, Lincoln, NE, USA) and processed using Image Studio software (LI-COR Biosciences).

### Reagents and antibodies, cytokines, and other reagents

The stock of CBR5884 (Sigma-Aldrich; SML1656), NCT-503 (Sigma-Aldrich; SML1659), bortezomib (Selleck Chemicals, Munich, Germany; S1013), and carfilzomib (Active Biochemicals CO, Wan Chai, Hong Kong; A-1098) were prepared in dimethyl sulfoxide (Sigma-Aldrich). Antibodies used were against PHGDH (Cell Signaling Technology, Danvers, MA, USA; CST #66350), PSAT1 (#PA5-22124), PSPH (#sc-271421), cleaved PARP (CST #5625), cleaved caspase-3 (CST #9664) and β-Actin (CST #4970). L-Serine (Sigma-Aldrich, MO, USA), Glycine (Millipore) and Minimum Essential Medium (MEM) (Gibco #21090) were used in serine starvation experiments.

### In vivo experiment

C57BL/KalwRij mice (Envigo Laboratories, Horst, Holland) were housed and treated according to procedures approved by the Ethical Committee for Animal Experiments of the Vrije Universiteit Brussel (project number: 19-281-7). Female mice were injected intravenously (IV) on day 0 with 5 × 10^5^ 5T33MM cells and divided into four groups: vehicle, bortezomib, NCT-503, and combination. Starting from day 1, 40 mg/kg NCT-503 treatment was given 6×/week intraperitoneally (IP) for the NCT-503 groups. Starting from day 2, 0.6 mg/kg bortezomib was given twice per week subcutaneously (SC). Injection volumes did not exceed 100 μl. The vehicle is composed of 5% ethanol, 35% PEG, and 60% of an aqueous 30% hydroxypropyl-â-cyclodextrin solution, and saline. At day 18, spleens were harvested and weighed, the percent plasma cells in the BM was determined on May Grunwald-Giemsa stained BM smears from one femur, and the M spike was measured as described [25].

### Statistical analysis

GraphPad Prism software version 8.0.0 (San Diego, CA, USA) was used for statistical analysis and generating figures. Non-linear regression analysis was used to fit the dose–response curves and to calculate IC50 values based on the variable slope model. Statistical significance was tested by extra sum-of-squares F test for IC50 values comparison of two groups, and two-way analysis of variance (ANOVA) followed by Bonferroni post-test for comparing GSH and NADPH/NADP^+^ measurements between INA6-KD cells. Unpaired t test was used to calculate the statistical differences of data from the in vivo experiment.

## Results

### MM cell lines express PHGDH and are sensitive to its inhibition

**Fig. 1:**
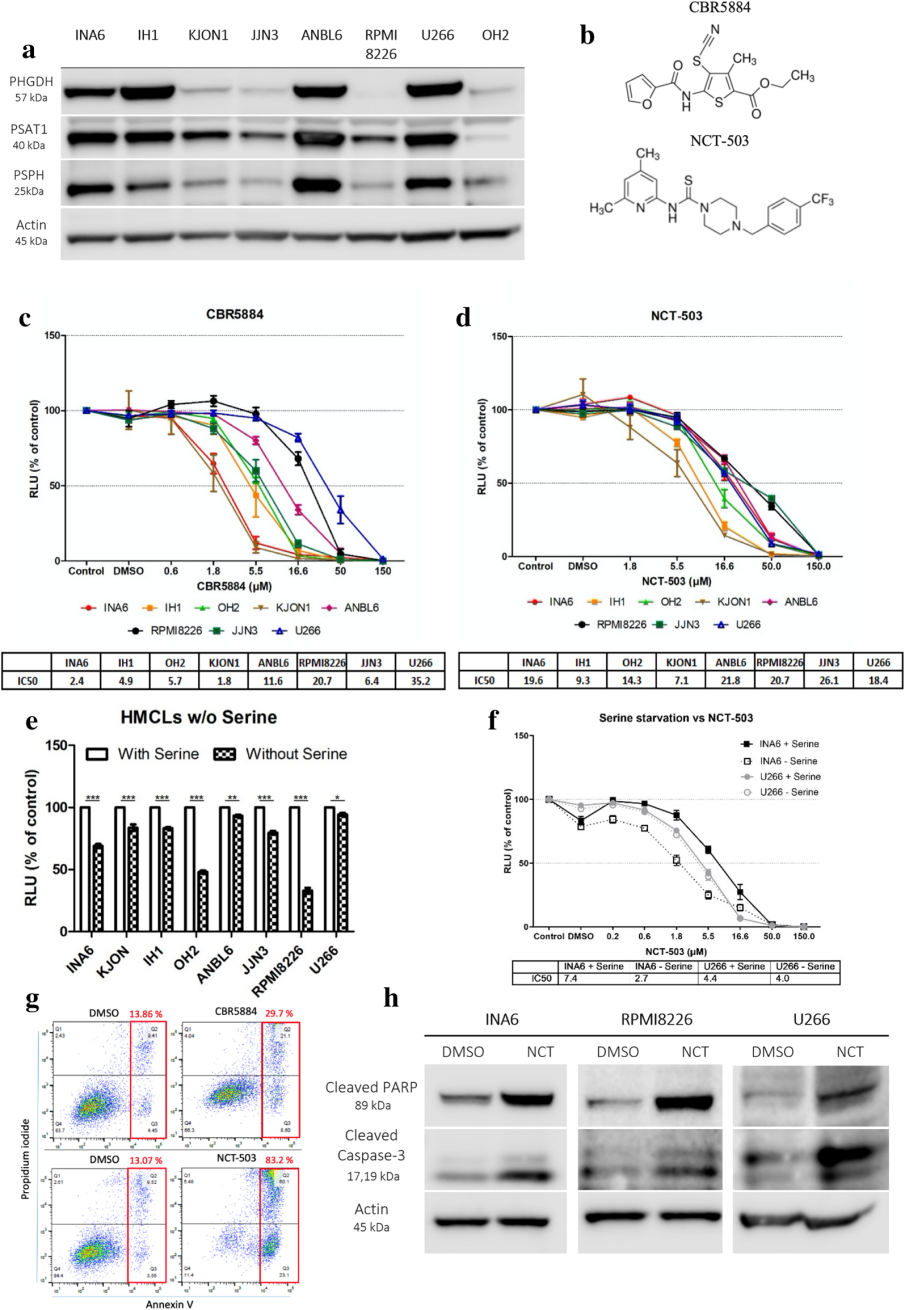
HMCLs express PHGDH and are sensitive to its inhibition. **a** The expression of PHGDH, PSAT1, and PSPH was assessed by WB in eight HMCLs. **b** The chemical structure of both PHGDH inhibitors, CBR5884 and NCT-503. **c**, **d** CellTiter-Glo was used to assess HMCLs’ sensitivity to both PHGDH inhibitors, CBR5884 and NCT-503, respectively. The cells were treated with indicated doses overnight. **e** Proliferation of HMCLs after an overnight incubation were incubated overnight with or without serine. **f** Proliferation of INA6 and U266 cells were treated overnight with different doses of NCT-503 in the presence or absence of serine. **g** INA6 cells were treated overnight with their corresponding IC50 × 3 doses of CBR5884 or NCT-503, and then apoptosis was assessed by flowcytometry using Annexin-V/PI assay. **h** INA6, RPMI8226 and U266 cells were treated for 4 h with double their corresponding IC50 of NCT-503, and then apoptotic proteins were investigated by WB. All the presented graphs and calculated IC50s represent three independent experiments with minimum two replicates. Error bars are ± SEM and *P ≤ 0.05*,* **P ≤ 0.01, ***P ≤ 0.001

To assess the role of SSP in our MM cell lines, we used Western blot (WB) and investigated the expression of the first three enzymes in this pathway, PHGDH, phosphoserine aminotransferase 1 (PSAT1), and phosphoserine phosphatase (PSPH) (Fig. 1a). The three enzymes seemed to be correlated in their expression and had very high protein levels in INA6, IH1, ANBL6, and U266. This high expression in some cells masked the weaker expressions by the rest, nonetheless, the three enzymes were expressed by all the tested MM cell lines.

Since PHGDH is the first and rate-limiting enzyme in this pathway, we tested the sensitivity of these cell lines to two PHGDH inhibitors with different modes of action, CBR5884 and NCT-503 (Fig. 1b). CBR5884 disrupt the oligomerization of PHGDH subunits, while NCT-503 decreases the melting temperature of PHGDH [26, 27]. Using CTG, an adenosine triphosphate (ATP)-dependent proliferation assay, all the cell lines were sensitive to both inhibitors, with CBR5884 generally being more potent than NCT-503 based on the calculated IC50s (Fig. 1c, d). Furthermore, to see whether high PHGDH expression made the cells independent of exogenous serine, we deprived them of serine overnight (Fig. 1e). Using the densitometry data generated from the WB (Fig. 1a), the MM cells lines showed a negative correlation (Spearman correlation: − 0.72; P value: 0.052) between PHGDH expression and sensitivity to serine starvation. However, the PHGDH expression and sensitivity to serine deprivation did not significantly correlate with the sensitivity of the cell lines to either of the inhibitors. Afterward, we hypothesized that serine starvation may sensitize the cells to PHGDH inhibition. To that end, we treated serine-starved INA6 and U266 cell with NCT-503. Serine starvation sensitized INA6 cells to NCT-503 treatment, but not U266 cells (Fig. 1f), suggesting that the sensitization may vary between cells based on their dependence on exogenous serine.

To compare the inhibitors’ ability to induce apoptosis, we treated INA6 cells overnight with 3 times IC50 of two inhibitors and ran the Annexin V/propidium iodide apoptosis assay. NCT-503 was more potent than CBR5884 in inducing apoptosis (Fig. 1g). To confirm that NCT-503 is mediating its effects through apoptosis, cleaved Poly(ADP-Ribose) polymerase (PARP) and cleaved caspase-3, which are early indicators of apoptosis, were assessed by WB. NCT-503-treated INA6, U266, and RPMI8226 had higher levels of cleaved PARP and caspase-3 than their DMSO-treated controls (Fig. 1h).

### Primary MM cells are sensitive to doses of the PHGDH inhibitor NCT-503, that are tolerated by PBMCs

**Fig. 2:**
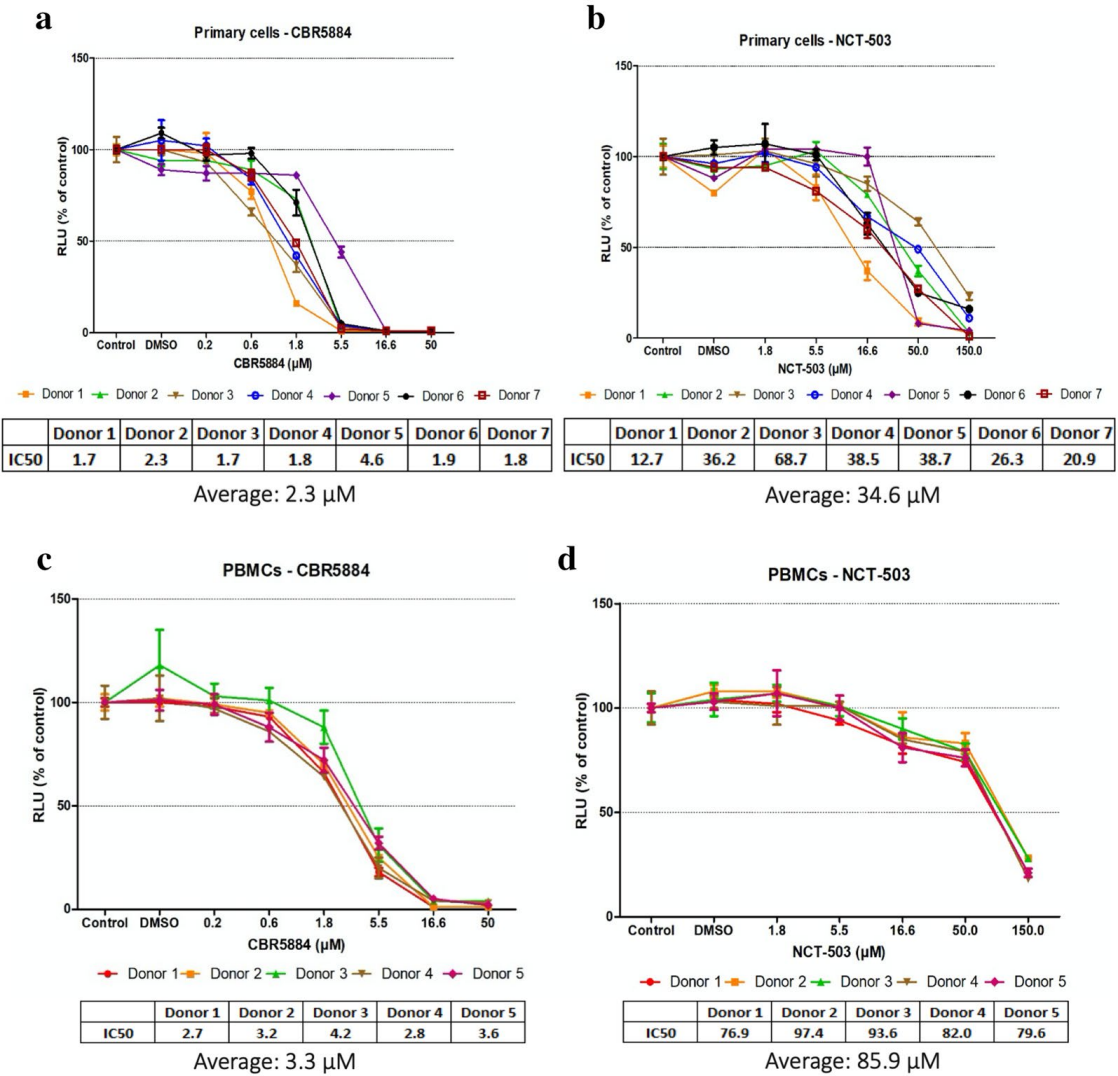
PHGDH inhibitors’ effects on proliferation of primary myeloma cells and PBMCs. CTG was used to assess the sensitivity to the inhibitors. **a**, **b** Primary cells treated overnight with indicated doses of CBR5884 and NCT-503, respectively. **c**, **d** PBMCs treated overnight with indicated doses of CBR5884 and NCT-503, respectively. The graphs and calculated IC50 values represent at least two technical replicates and the error bars are ± SD

To assess the therapeutic potential of targeting PHGDH with CBR5884 and NCT-503, we tested the inhibitors against primary MM cells from patients and PBMCs from healthy donors. When comparing the sensitivity of primary cells and PBMCs, no significant difference was seen upon CBR5884 treatment (average 2.3 μM vs 3.3 μM) (Fig. 2a, c), whereas their sensitivity to NCT-503 (Fig. 2b, d) was noticeably different with the primary cells being sensitive to doses of the inhibitor that were tolerated by PBMCs (average 34.6 μM vs 85.9 μM). This highlights the potential of using NCT-503 as a therapeutic molecule to target PHGDH in MM.

### Synergy between PHGDH inhibitors and bortezomib

Since SSP was reported as a contributor to bortezomib resistance [13], we hypothesized that inhibiting PHGDH would potentiate the effect of bortezomib and possibly other PIs, such as carfilzomib. To test our hypothesis, we treated each of the eight MM cell lines with carfilzomib or bortezomib dilutions in the presence or absence of PHGDH inhibitors. The cells were treated with 1/3 of their respective IC50 doses for CBR5884 and NCT-503 to be sure that the effect of the PHGDH inhibitors was not too strong on its own. Furthermore, we normalized the data to the controls (without carfilzomib or bortezomib treatment) of each condition (presence or absence of PHGDH inhibitor). We did the latter to eliminate the additive effect of PHGDH inhibitors and investigate the synergy with the PIs. To make the interpretation of those results easier (individual experiments are presented in Additional file 1: Fig. S1A–C), the data were summarized into tables based on the IC50 values, and the fold change in the PI’s IC50 was calculated (Table 2 A and B). Surprisingly, the two inhibitors did not show the same trends. CBR5884 significantly potentiated the effects of both carfilzomib and bortezomib mainly on four cell lines, JJN3, RPMI8226, ANBL6, and U266, while it only potentiated the effects of carfilzomib on IH1. NCT-503 significantly potentiated the effect of bortezomib on all the tested cell lines, except for JJN3, but did not potentiate the effect of carfilzomib.

**Table 2:** Synergy experiments with HMCLs

A						B					
CBR5884						NCT-503					
Cells	Proteosome inhibitor	IC50 of PI		Fold change in IC50	*P* value	Cells	Proteosome inhibitor	IC50 of PI		Fold change in IC50	*P* value
		- CBR	+ CBR					- NCT	+ NCT		
INA6	Carf	1.2	1.1	0.90	0.5074	INA6	Carf	1.2	1.2	0.98	0.8862
	BTZ	14.6	16.5	1.13	0.0406		BTZ	15.4	8.6	0.56	< 0.0001
KJON1	Carf	2.4	2.1	0.88	0.1246	KJON1	Carf	2.7	3.3	1.22	0.0186
	BTZ	27.4	24.6	0.90	0.1895		BTZ	26.1	19.3	0.74	0.0001
IH1	Carf	5.2	4.1	0.79	0.0058	IH1	Carf	5.6	6.4	1.15	0.0408
	BTZ	14.7	12.6	0.86	0.1743		BTZ	12.7	11.1	0.88	0.0258
OH2	Carf	2.5	2.2	0.90	0.3532	OH2	Carf	2.6	2.6	1.01	0.9351
	BTZ	11.0	9.2	0.84	0.0472		BTZ	11.5	6.5	0.56	< 0.0001
JJN3	Carf	3.7	2.1	0.58	< 0.0001	JJN3	Carf	3.7	5.8	1.59	< 0.0001
	BTZ	17.4	11.4	0.66	< 0.0001		BTZ	16.7	15.4	0.92	0.2634
RPMI8226	Carf	3.2	1.5	0.47	< 0.0001	RPMI8226	Carf	3.0	3.4	1.14	0.2898
	BTZ	17.6	11.6	0.66	< 0.0001		BTZ	16.8	10.5	0.62	< 0.0001
ANBL6	Carf	6.4	4.3	0.67	< 0.0001	ANBL6	Carf	5.7	7.8	1.36	0.0003
	BTZ	21.5	14.7	0.69	0.0008		BTZ	21.5	15.2	0.71	0.0143
U266	Carf	5.6	3.2	0.57	< 0.0001	U266	Carf	6.7	7.3	1.10	0.458
	BTZ	23.0	13.3	0.58	< 0.0001		BTZ	23.4	12.6	0.54	< 0.0001

HMCLS were treated with 1/3 of their corresponding IC50 of either A) CBR5884 (CBR) or B) NCT-503 (NCT), in combination with either carfilzomib (Carf) or bortezomib (BTZ). The table summarizes the proteasome inhibitors’ (PI) IC50 values obtained from the graphs shown in Additional file 1: Fig. S1A, B and C

### PHGDH knockdown recapitulated the results seen with NCT-503 in INA6

**Fig. 3:**
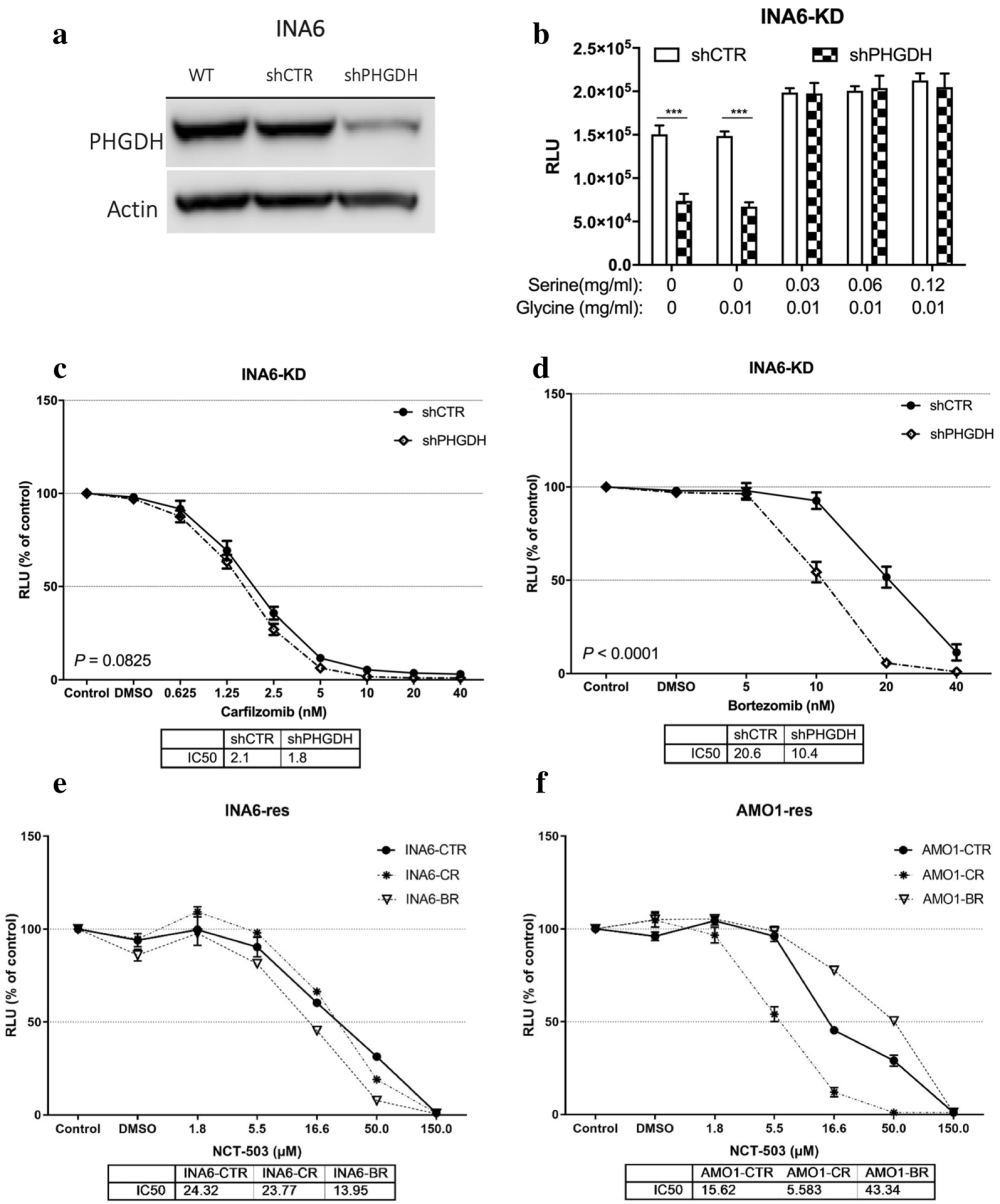
PHGDH knockdown potentiates bortezomib and proteasome-resistant cell lines are sensitive to PHGDH inhibition. **a** The knockdown of PHGDH using shRNA was confirmed via WB. **b** CTG was used to highlight the dependence of the shPHGDH cells on extracellular serine. The sensitivity to drugs was tested by CTG. **c**, **d** INA6 KD cells were treated overnight with carfilzomib and bortezomib, respectively. **e**, **f** INA6-res and AMO1-res cell lines, respectively, were treated with the indicated doses of NCT-503 overnight. All the presented graphs and calculated IC50s represent three independent experiments with minimum two replicates. Error bars are ± SEM and *P ≤ 0.05*,* **P ≤ 0.01, ***P ≤ 0.001

To validate the results obtained with the inhibitors, INA6 cells were transduced with non-target control shRNA or shRNA targeting PHGDH. INA6 was a good candidate for the transduction due to the discrepancy between the two PHGDH inhibitors in potentiating the effect of bortezomib in this cell line. The knockdown was validated by WB (Fig. 3a). To see whether the reduction in PHGDH level made the cells more dependent on exogenous serine, INA6-KD were incubated overnight in media lacking serine and glycine, and then treated with increasing doses of serine (Fig. 3b). As anticipated, shPHGDH cells were more vulnerable to serine deprivation than control cells but they were completely rescued by 0.03 mg/ml of exogenous serine (corresponding to the serine levels found in RPMI1640 media). On the other hand, exogenous glycine could not compensate for the lack of serine.

Next, the knockdown cells were tested for their sensitivity to the proteasome inhibitors. The knockdown of PHGDH imitated the effect of NCT-503 treatment on INA6 cells by potentiating the effect of bortezomib but not carfilzomib (Fig. 3c, d), suggesting that the observed effect of NCT-503 is due to on-target inhibition of PHGDH.

### Proteasome inhibitor-resistant cell lines are sensitive to PHGDH inhibition

We questioned if inhibiting PHGDH in cell lines resistant to PIs could resensitize them. To that end, we used two PI-resistant cell lines, INA6-res and AMO1-res. Each cell line has three clones, CTR, carfilzomib-resistant (CR), and bortezomib-resistant (BR). First, the IC50 dose of NCT-503 was determined for each of the resistant cell lines (Fig. 3e, f). Both PI-resistant cell lines were sensitive to NCT-503 treatment. Interestingly, AMO1-CR were more sensitive than AMO1-CTR and AMO1-BR counterparts. Whereas for the INA6-res, INA6-BR was the most sensitive. An estimation of the PHGDH protein levels in these cells using WB showed no apparent correlation with their sensitivity to the PHGDH inhibitors (Additional file 2: Fig. S2A, B). Finally, the synergy between NCT-503 and the PIs was tested on both resistant cell lines (Additional file 3: Fig. S3A, B), and the results were summarized in a table (Table 3). NCT-503 treatment made all INA6-res and AMO1-res clones more sensitive to bortezomib, but made only INA6-CR more sensitive to carfilzomib.

**Table 3:** Synergy experiments with PI-resistant cell lines

	NCT-503					
	Cells	Proteosome inhibitor	IC50 of PI		Fold change in IC50	*P* value
			- NCT	+ NCT		
INA6-res	CTR	Carf	3.6	3.2	0.89	0.1404
		BTZ	22.7	15.0	0.66	< 0.0001
	CR	Carf	12.0	7.0	0.58	< 0.0001
		BTZ	46.6	25.8	0.55	< 0.0001
	BR	Carf	3.2	2.9	0.91	0.268
		BTZ	59.0	34.3	0.58	< 0.0001
AMO1-res	CTR	Carf	2.4	2.2	0.92	0.1998
		BTZ	24.4	7.2	0.30	< 0.0001
	CR	Carf	711.0	645.0	0.91	0.1521
		BTZ	199.0	113.0	0.57	< 0.0001
	BR	Carf	25.0	27.0	1.08	0.4697
		BTZ	1111.0	581.0	0.52	< 0.0001

INA6-res and AMO1-res cells were treated with 1/3 of their corresponding IC50 of NCT-503 (NCT), in combination with either carfilzomib (Carf) or bortezomib (BTZ). The table summarizes the proteasome inhibitors’ (PI) IC50 values obtained from the graphs shown in Additional file 3: Fig. S3a, b.

### PHGDH inhibition reduces the redox capacity of the cells

**Fig. 4:**
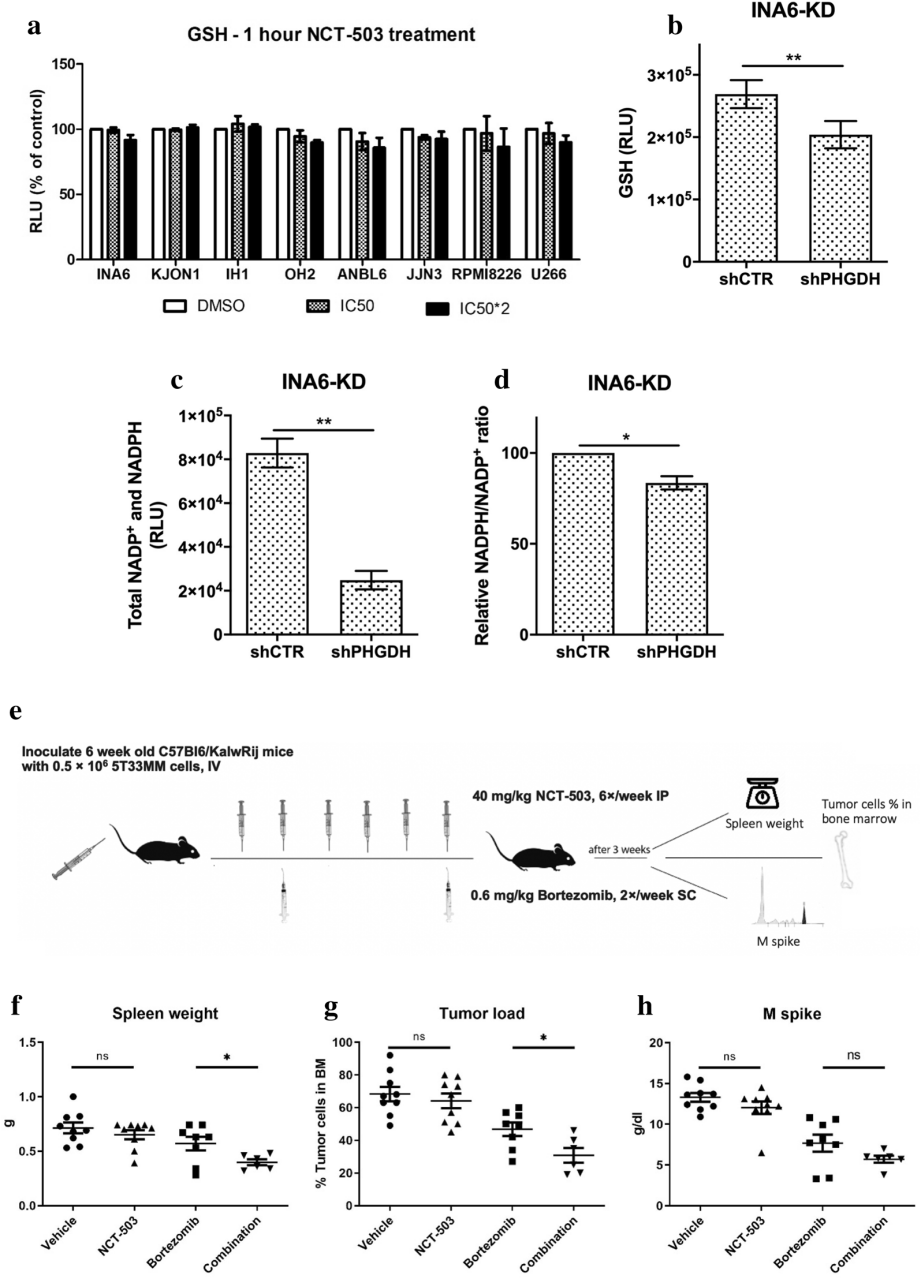
PHGDH inhibition reduces the intracellular redox capacity and gives a therapeutic advantage in vivo*.* **a** HMCLs were treated overnight with their corresponding NCT-503 IC50 or double the IC50 (IC50*2), and then GSH levels were measured. **b** GSH levels, **c** total NADP^+^ and NADPH, and **d** relative NADPH/NADP^+^ ratio in untreated INA6-KD cells was measured. Graph A represents two independent experiments while graphs B-D represent three independent experiments, all with minimum two replicates. **e** Schematic image of the experimental setup using the 5T33MM model. The mice were divided into four groups: vehicle (n = 9), NCT-503 (n = 9), bortezomib (n = 8), and combination (n = 6). **f** Spleens were weighed. **g** BMs were harvested for analysis of plasmacytosis on cytosmears. **h** M spike was analyzed by protein electrophoresis on collected serum. Error bars are ± SEM and *P ≤ 0.05*,* **P ≤ 0.01, ***P ≤ 0.001

SSP is important for GSH synthesis. GSH is a key antioxidant that protects against oxidative stress, and it requires NADPH to regenerate its reduced state and maintain the redox homeostasis in cells [10, 28]. As previous studies have linked the cytotoxicity of bortezomib in MM to the intercellular levels of GSH [24], we hypothesized that NCT-503 could have an effect on the redox capacity of the cells. Knowing that the half-life of GSH is 10 min [29], NCT-503 treatment for 1 h was expected to be sufficient to see an effect on the GSH level. To avoid any cell-death-mediated drop in the GSH measurements, we ensured that the tested MM cell lines can survive a 1-h treatment of twice their respective IC50 dose of NCT-503 (Additional file 4: Fig. S4A). Although several of the tested cell lines showed a trend towards decreased levels of GSH, the decrease was marginal (Fig. 4a). Repeating the experiments with a 4-h treatment of 1/3 of the IC50 caused a similar marginal decrease (results not shown). Nevertheless, knockdown of PHGDH in INA6 cells significantly reduced the levels of GSH (Fig. 4b), and it reduced the total levels of NADPH + NADP^+^ and the NADPH/NADP^+^ ratio (Fig. 4c, d).

### In vivo experiment

To assess the therapeutic potential of targeting PHGDH in vivo by NCT-503, alone and in combination with bortezomib, a murine myeloma model was used. C57BL/KaLwRij mice were injected with 5T33MM cells and then divided into four groups to receive either NCT-503, bortezomib, or both, in addition to a control group treated with the drug vehicle alone (Fig. 4e). The 5T33MM model is an immune-competent mouse model that recapitulates the human disease by secreting M-proteins, residing in the BM, and migrating to hematopoietic organs including the spleen [30]. While single treatment with 40 mg/kg NCT-503 had no or marginal effect on tumor burden in any of the parameters measured, it could potentiate the effect of low doses (0.6 mg/kg) of bortezomib. Spleen size was significantly reduced by an extra 32% in the combo group compared to single bortezomib (mean 0.39 g vs 0.57 g). When examining M spike and tumor load in the BM, we found an extra reduction of 25% (5.7 g/dl vs 7.6 g/dl) and 35% (30% vs 46%), respectively (Fig. 4f–h). This indicates that combining bortezomib with inhibition of PHGDH can be beneficial in treatment of MM. In a pilot experiment, treating three mice with NCT-503 only at a dose of 80 mg/kg indicated a clinical effect of NCT-503 alone, but had severe side-effects (result not shown).

## Discussion

Cancer cells can adjust their needs to the limited and sometimes harsh tumor microenvironments by metabolic adaptations [7]. PHGDH is overexpressed in many cancers, either via gene copy-number gain, or transcriptional upregulation mediated via activation of transcription factor 4 (ATF4). SSP is necessary to provide cancer cells with essential amino acids for cell survival and proliferation [11, 31, 32]. Moreover, PHGDH is upregulated in MM compared to two related B cell malignancies [8], which encouraged us to target PHGDH with two different small molecular inhibitors.

Treating HMCLs with either NCT-503 or CBR5884 reduced proliferation. However, the two inhibitors had different potency on the tested cell lines. All cell lines expressed PHGDH, but the level of expression varied. The expression levels did not correlate with the sensitivity to either of the inhibitors. One possible explanation for this discrepancy is the cells’ capability to import serine. Hence, we checked for the sensitivity of the HMCLs to serine starvation as an indicator of the dependency of these cells on external serine for proliferation. The cell lines’ expression of PHGDH was inversely proportional to their sensitivity to serine starvation. Yet again, we found no direct correlation between the sensitivity to serine starvation and the sensitivity to either of the inhibitors. Another possible contributor to the discrepancy could be the difference in the cellular redox state between these cells. In a recent study, the cellular redox state was found to constrain SSP through regulation of the availability of the co-factor NAD^+^ needed for PHGDH enzymatic activity [33]. Moreover, some of the variations could be explained by PHGDH having a more promiscuous role outside the SSP. Recently, PHGDH was reported to reduce α-ketoglutarate (αKG) to _D-_2-hydroxyglutarate (_D_-2HG) [34]. _D_-2HG is an oncometabolite that serves as a competitive inhibitor of αKG-dependent enzymes, such as histone demethylases, and is could promote leukemogenesis [35–37].

The two PHGDH inhibitors have different mechanisms of action, hence this may explain the difference between the two in spite of targeting the same protein [26, 27]. NCT-503 exhibited a possible therapeutic advantage over CBR5884 as primary MM cells were sensitive to doses of NCT-503 that were tolerated by PBMCs from healthy donors, whereas CBR5884 was equally harmful to both. When treated with three times the calculated IC50 from the CTG assay, NCT-503 was more potent than CBR5884 in inducing apoptosis. This suggests that in INA6 cells NCT-503 is cytotoxic whereas CBR5884 is mainly cytostatic at the used doses. Furthermore, knockdown of PHGDH in INA6 cells mimicked the sensitizing effect of NCT-503 in combination treatment with bortezomib on INA6 cells but did not mimic the effect of CBR5884. This suggested that NCT-503 is a promising therapeutic agent to be used for targeting PHGDH. However, the possibility of off target effects cannot be ruled out.

Unlike the NCT-503 treatment, the stable knockdown of PHGDH seemed not to have an effect on INA6 survival in the presence of exogenous serine. The latter could be due to the knockdown reflecting a low-dose treatment of NCT-503 that may not affect survival, but enough to drive the synergy with bortezomib. Furthermore, the stable knockdown of PHGDH would become a selective survival pressure favoring the survival of cells able to survive the reduced PHGDH levels.

Our results showing synergy between NCT-503 and bortezomib are in line with the previous study attributing reduced bortezomib cytotoxicity to upregulated SSP in MM [13]. Knowing that both carfilzomib and bortezomib inhibit proteasomes, it is unclear what makes NCT-503 potentiate only bortezomib. Nonetheless, the PI-resistant cell lines were sensitive to NCT-503 treatment. Furthermore, NCT-503 treatment significantly resensitized INA6-res and AMO1-res cells to bortezomib, and to carfilzomib in INA6-CR. These results highlight the potential benefit of the combination treatment, especially in patients who develop bortezomib resistance.

In addition to its role in bortezomib resistance in MM, SSP’s was also found to contribute to the resistance against BRAF inhibitors used to treat cancers expressing V600E BRAF mutation, such as melanoma, pancreatic and non-small cell lung cancer cells [38]. Similarly, resistance to tyrosine kinase inhibitors (TKIs) in hepatocellular carcinoma and EGFR mutation-positive lung adenocarcinomas was counteracted by PHGDH knockdown. NCT-503 treatment also synergized with the TKIs in a similar manner to our results with bortezomib [39, 40]. In line with the above-mentioned studies, our findings suggest that the effects seen by targeting PHGDH are partially mediated by reducing the intracellular redox capacity. This is achieved by reducing the levels of GSH, total NADPH and NADP^+^, and NADPH/NADP^+^ ratio.

In our mouse model, NCT-503 treatment did not reduce tumor load at the doses used but reduced tumor growth in combination with bortezomib compared to bortezomib alone. This suggests that NCT-503 was active in mice but had no direct cytotoxic effects on its own with the dose used. The absence of NCT-503-induced cytotoxicity could be due to in vivo interactions that either weakens the inhibitors effect, or makes the MM cells more resistant. However, the dose of NCT-503 treatment was strong enough to potentiate the effect of bortezomib when used in combination. During the preparation of this manuscript, a publication by Wu et al*.* confirmed our results (41). They showed that overexpressing PHGDH promotes proliferation and bortezomib resistance through increasing GSH synthesis in MM. In their xenograft model tumor cells overexpressing PHGDH were more resistant to bortezomib treatment. The latter fully supports our data which demonstrates the therapeutic potential of inhibiting PHGDH in combination with bortezomib.

## Conclusions

Our study is the first to target PHGDH in an in vivo model of MM, thereby further underscoring the importance and dependency of MM cells on the SSP. This pathway is reported to contribute to drug resistance in different malignancies, including bortezomib resistance in MM. Our results demonstrate the therapeutic potential of targeting PHGDH specifically in combination with bortezomib. A better understanding of the involved mechanism may help to develop more efficient combinational treatments in the future.

## Supplementary Information

**Additional file 1: Fig. S1.** Synergy experiments between NCT-503 and BTZ in HMCLs. CTG was used to assess the synergic effect. HMCLs were treated with 1/3 of their corresponding IC50 of either CBR5884 or NCT-503, in combination with either carfilzomib or bortezomib. A) INA6, KJON1, and IH1. B) OH2, ANBL6, and U266. C) JJN3 and RPMI. The graphs represent three independent experiments with minimum two replicates. Error bars are ± SEM.

**Additional file 2: Fig. S2.** PHGDH expression in proteasome-resistant cell lines. A) and B) PHGDH expression in INA6-res and AMO1-res cell lines, respectively, was assessed via WB.

**Additional file 3: Fig. S3.** Synergy experiments between NCT-503 and BTZ in proteasome-resistant cell lines. CTG was used to assess the synergic effect. A) INA6 res. and B) AMO1 res. cell lines were treated with 1/3 of their corresponding IC50 of either CBR5884 or NCT-503, in combination with either carfilzomib or bortezomib. The graphs represent three independent experiments with minimum two replicates. Error bars are ± SEM.

**Additional file 4: Fig. S4.** Cell viability was not affected by NCT-503 treatment for 1 h. Annexin V/PI assay was used to determine cell viability. HMCLs were treated for 1 h with double of their corresponding IC50 of NCT-503. The graphs represent two independent experiments with minimum two replicates. Error bars are ± SEM.

## Data Availability

Data sharing is not applicable to this article as no datasets were generated or analysed during the current study.

## Abbreviations

MM: Multiple myeloma
BM: Bone marrow
IMIDs: Immune modulators
PIs: Proteasome inhibitors
PHGDH: Phosphoglycerate dehydrogenase
GSH: Glutathione
HMCLs: Human myeloma cell lines
PBMCs: Peripheral blood mononuclear cells
KD: Knockdown
shCTR: Cells transduced with lentiviral particles containing non-target control shRNA
shPHGDH: Cells transduced with lentiviral particles containing shRNA targeting PHGDH
CR: Carfizomib-resistant
BR: Bortezomib resistant
CTR: Control
CTG: CellTiter-Glo
RLU: Relative Luminescence Unit
PBS: Phosphate-buffered saline
IV: Intravenous
IP: Intraperitoneal
SC: Subcutaneous
ANOVA: Two-way analysis of variance
WB: Western blot
PSAT1: Phosphoserine aminotransferase 1
PSPH: Phosphoserine phosphatase
PARP: Poly(ADP-Ribose) polymerase
αKG: α-Ketoglutarate
_D_-2HG: _D-_2-hydroxyglutarate
TKIs: Tyrosine kinase inhibitors
HS: Heat-inactivated human serum
FCS: Fetal calf serum
