# Supplementary material for: Targeting phosphoglycerate dehydrogenase in multiple myeloma
Source: Exp Hematol Oncol. 2021 Jan 4;10:3. doi: 10.1186/s40164-020-00196-w (PMC7784327; doi:10.1186/s40164-020-00196-w)

Supplementary Fig.1

A

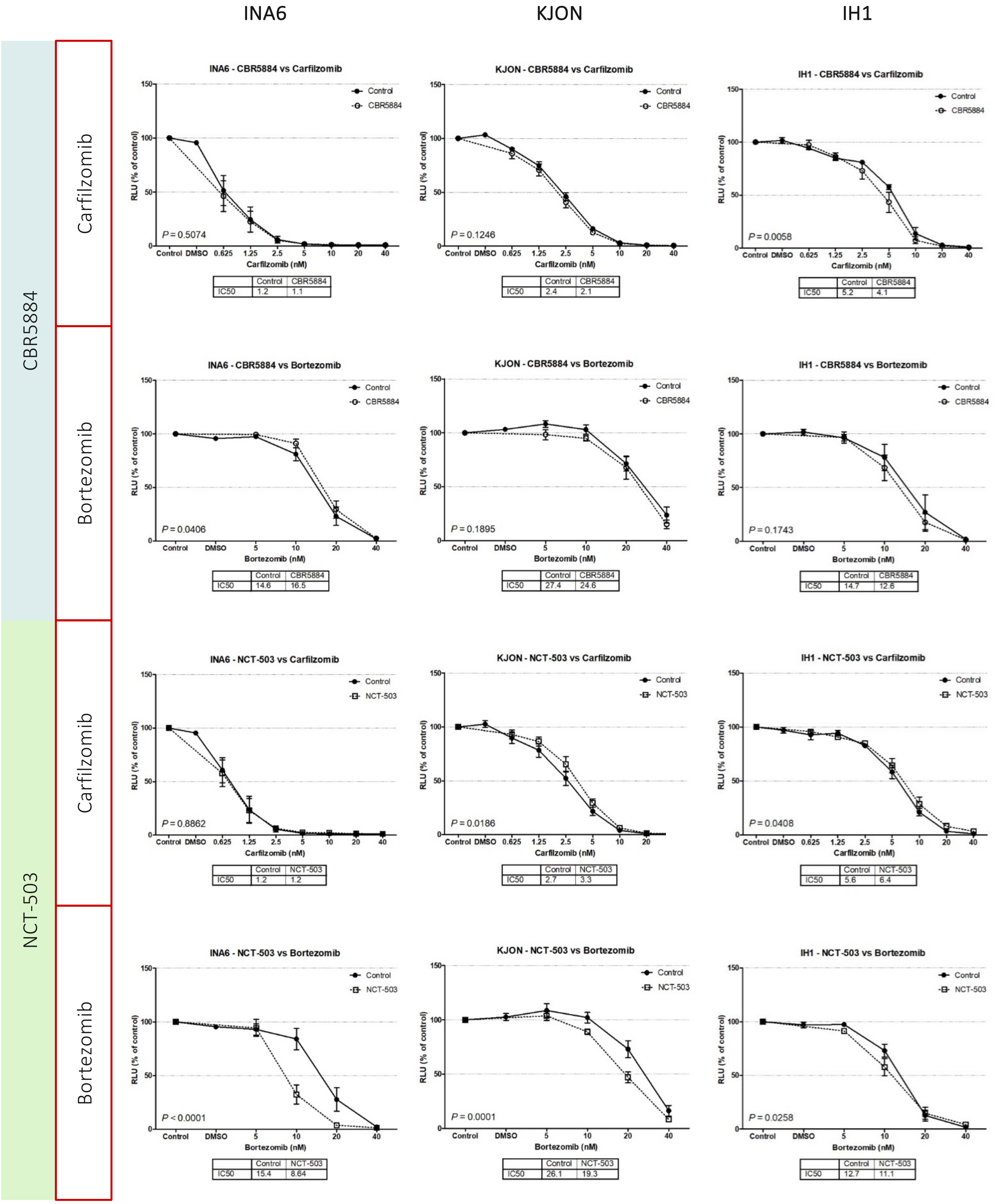

Supplementary Fig.1

B

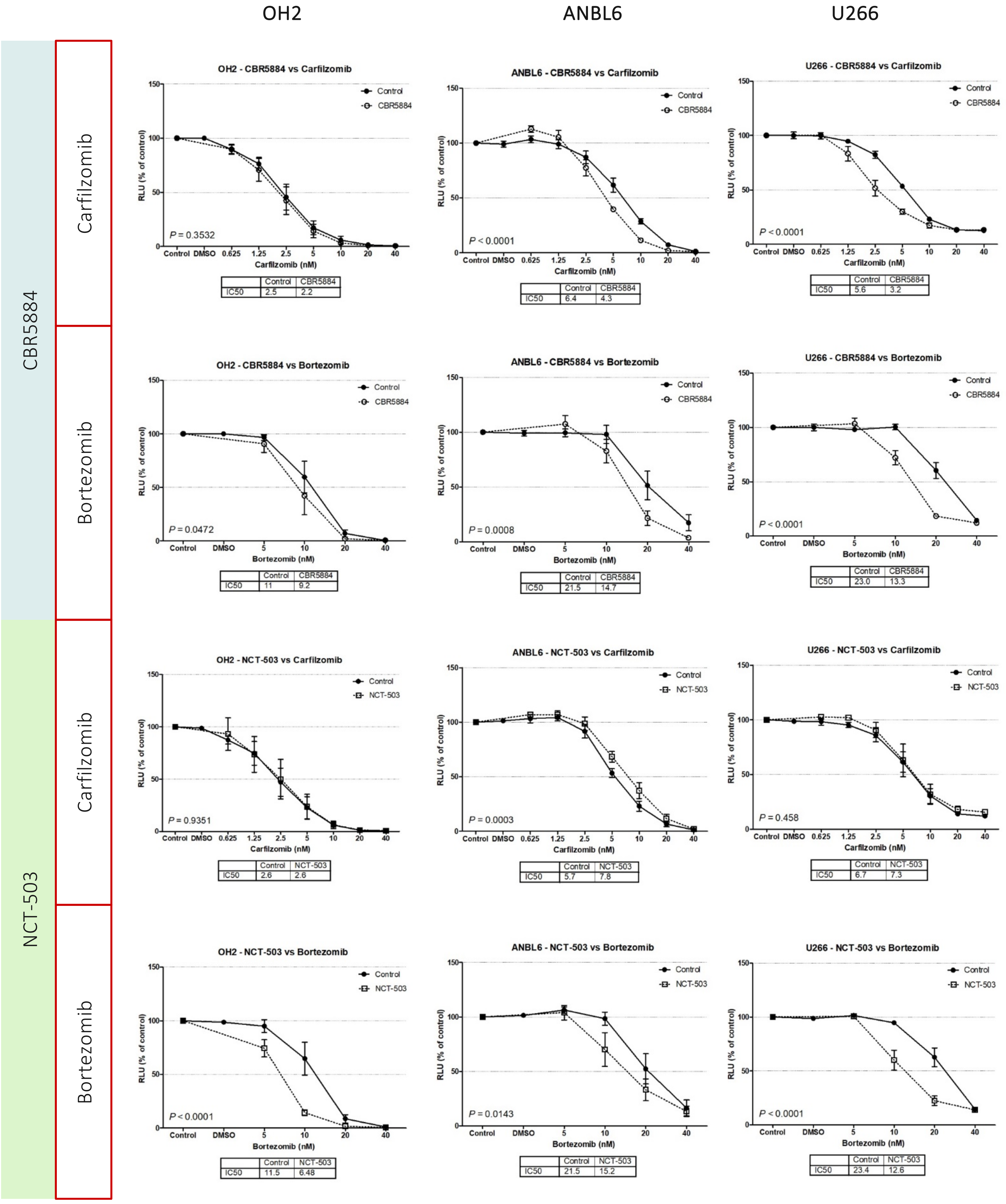

OH2 - CBR5884 vs Carfilzomib

	Control	CBR5884
IC50	2.5	2.2

ANBL6 - CBR5884 vs Carfilzomib

	Control	CBR5884
IC50	6.4	4.3

U266 - CBR5884 vs Carfilzomib

	Control	CBR5884
IC50	5.6	3.2

OH2 - CBR5884 vs Bortezomib

	Control	CBR5884
IC50	11	9.2

ANBL6 - CBR5884 vs Bortezomib

	Control	CBR5884
IC50	21.5	14.7

U266 - CBR5884 vs Bortezomib

	Control	CBR5884
IC50	23.0	13.3

OH2 - NCT-503 vs Carfilzomib

	Control	NCT-503
IC50	2.6	2.6

ANBL6 - NCT-503 vs Carfilzomib

	Control	NCT-503
IC50	5.7	7.8

U266 - NCT-503 vs Carfilzomib

	Control	NCT-503
IC50	6.7	7.3

OH2 - NCT-503 vs Bortezomib

	Control	NCT-503
IC50	11.5	6.48

ANBL6 - NCT-503 vs Bortezomib

	Control	NCT-503
IC50	21.5	15.2

U266 - NCT-503 vs Bortezomib

	Control	NCT-503
IC50	23.4	12.6

Supplementary Fig.1

c

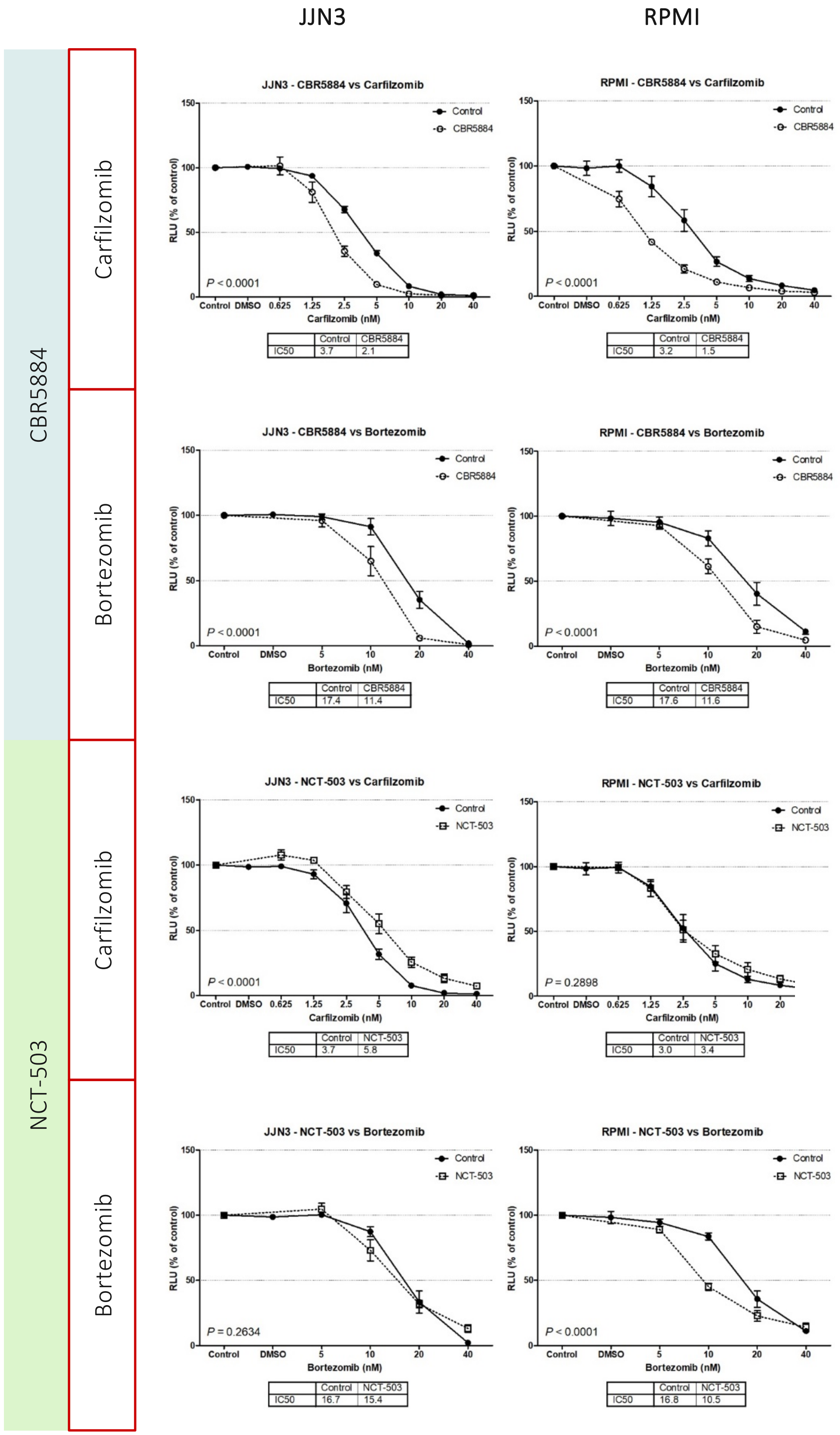

Supplement: Supplementary file 1 — Additional file 1: Fig. S1. Synergy experiments between NCT-503 and BTZ in HMCLs. CTG was used to assess the synergic effect. HMCLs were treated with 1/3 of their corresponding IC50 of either CBR5884 or NCT-503, in combination with either carfilzomib or bortezomib. A) INA6, KJON1, and IH1. B) OH2, ANBL6, and U266. C) JJN3 and RPMI. The graphs represent three independent experiments with minimum two replicates. Error bars are ± SEM. [file 40164_2020_196_MOESM1_ESM.pdf]
