# Supplementary figures and images for: Targeting phosphoglycerate dehydrogenase in multiple myeloma
Source: Exp Hematol Oncol. 2021 Jan 4;10:3. doi: 10.1186/s40164-020-00196-w (PMC7784327; doi:10.1186/s40164-020-00196-w)

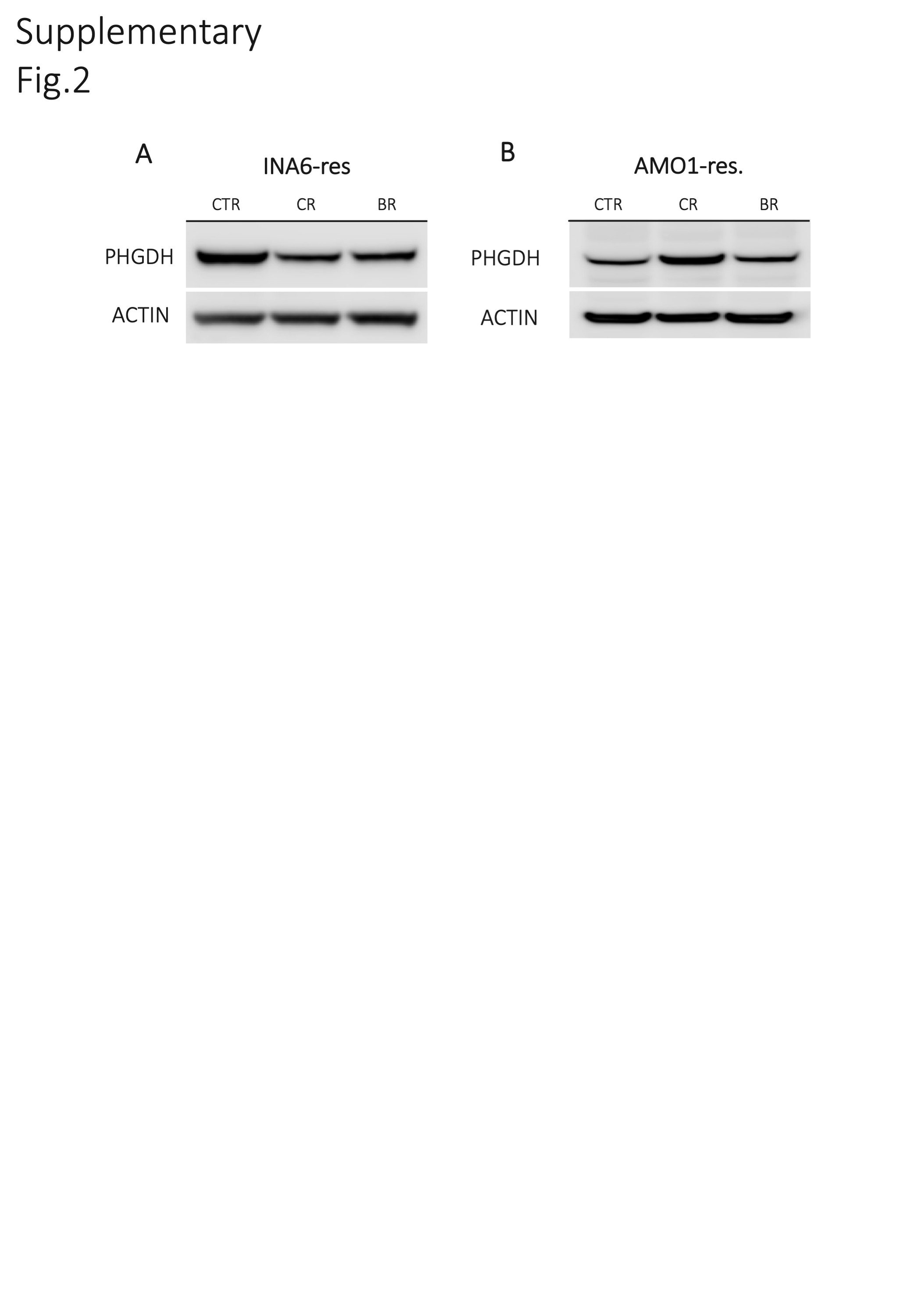

Supplement: Supplementary file 2 — Additional file 2: Fig. S2. PHGDH expression in proteasome-resistant cell lines. A) and B) PHGDH expression in INA6-res and AMO1-res cell lines, respectively, was assessed via WB. [file 40164_2020_196_MOESM2_ESM.tiff]

# Supplementary

## Fig.3

A

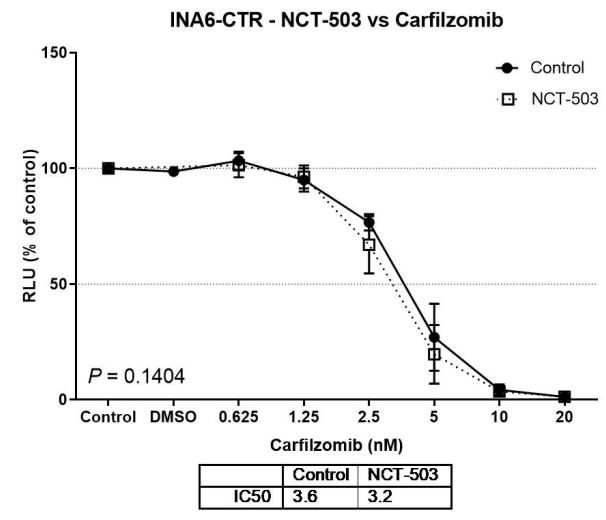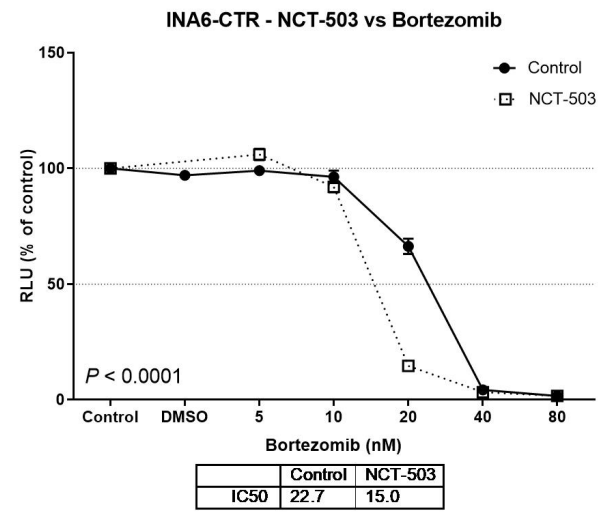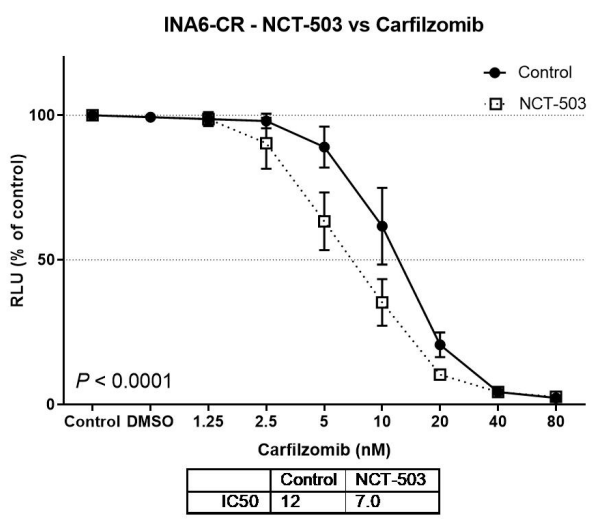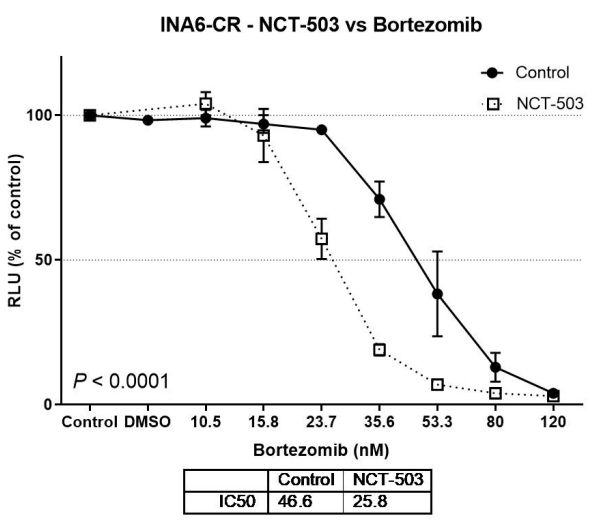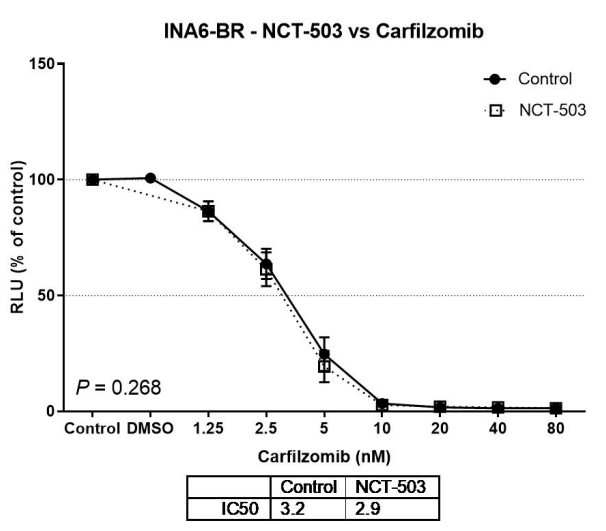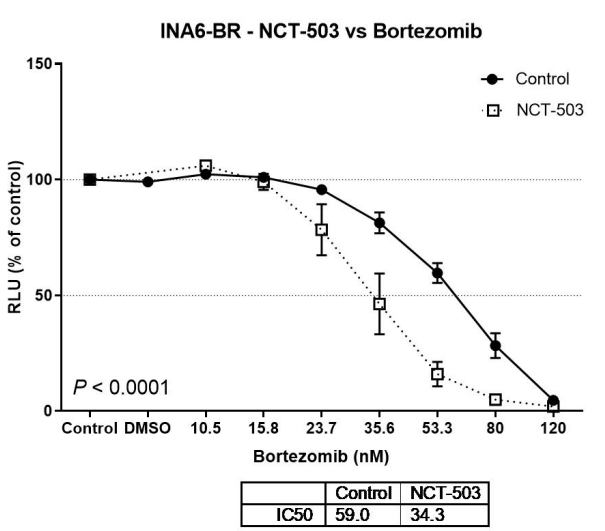

# Supplementary

## Fig.3

B

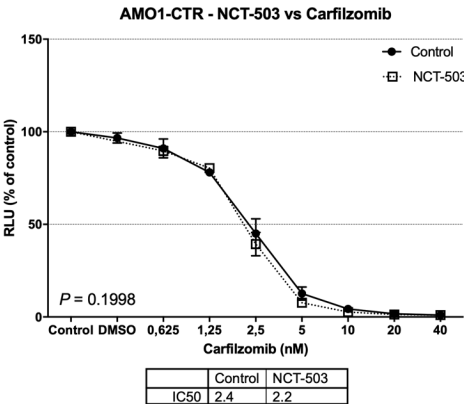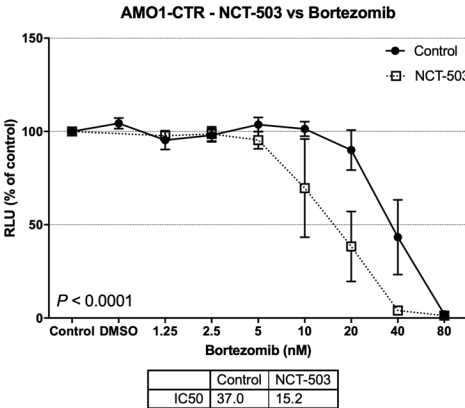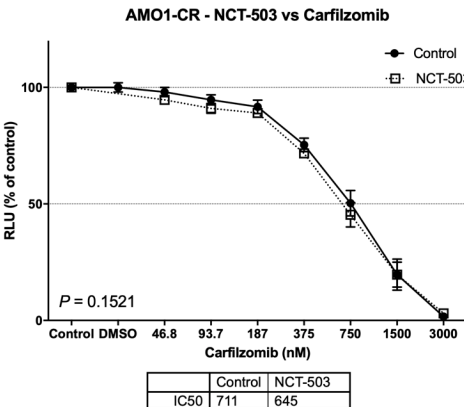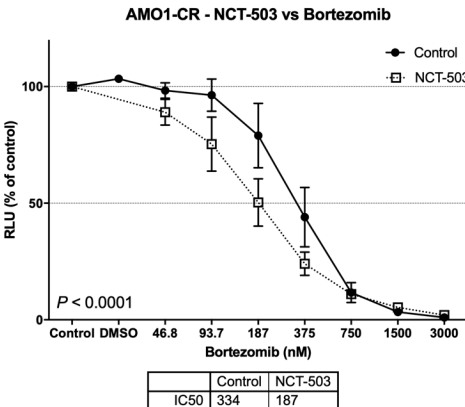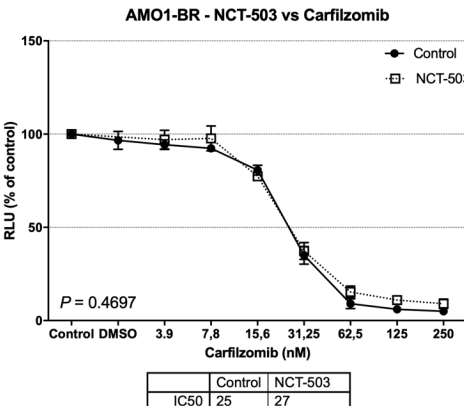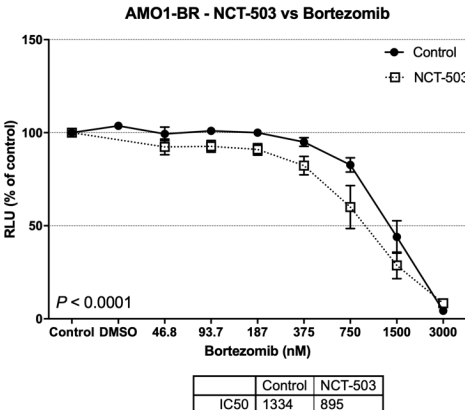

Supplement: Supplementary file 3 — Additional file 3: Fig. S3. Synergy experiments between NCT-503 and BTZ in proteasome-resistant cell lines. CTG was used to assess the synergic effect. A) INA6 res. and B) AMO1 res. cell lines were treated with 1/3 of their corresponding IC50 of either CBR5884 or NCT-503, in combination with either carfilzomib or bortezomib. The graphs represent three independent experiments with minimum two replicates. Error bars are ± SEM. [file 40164_2020_196_MOESM3_ESM.pdf]

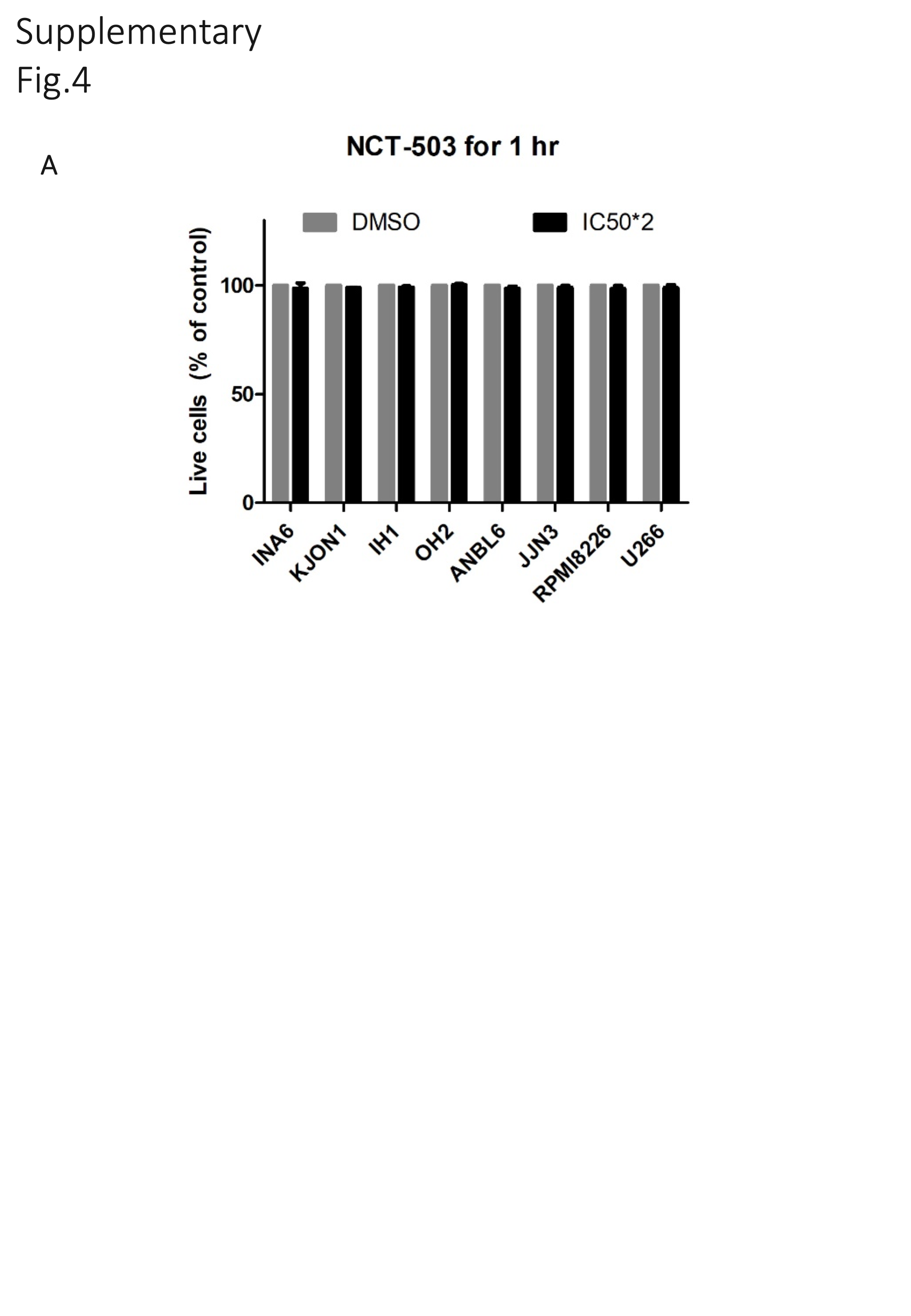

Supplement: Supplementary file 4 — Additional file 4: Fig. S4. Cell viability was not affected by NCT-503 treatment for 1 h. Annexin V/PI assay was used to determine cell viability. HMCLs were treated for 1 h with double of their corresponding IC50 of NCT-503. The graphs represent two independent experiments with minimum two replicates. Error bars are ± SEM. [file 40164_2020_196_MOESM4_ESM.tiff]
